# School-based caries prevention and longitudinal trends in untreated decay: an updated analysis with Markov chains

**DOI:** 10.1186/s13104-020-4886-8

**Published:** 2020-01-10

**Authors:** Ryan Richard Ruff, Deepak Saxena, Richard Niederman

**Affiliations:** 1 Department of Epidemiology & Health Promotion, New York University College of Dentistry, 433 First Avenue, Room 712, New York, NY 10010 USA; 2 New York University College of Global Public Health, New York, USA; 3 Department of Basic Science and Craniofacial Biology, New York University College of Dentistry, New York, USA

**Keywords:** Dental caries, Children, Adolescents, Schools, Oral health, Preventive medicine, Prevention

## Abstract

**Objective:**

Dental caries (tooth decay) is the most prevalent childhood disease in the world. A school-based program for the prevention of dental caries providing bi-annual sealants, interim therapeutic restorations, and fluoride varnish to children aged 5–12 years was previously associated with a significant reduction in the prevalence of untreated tooth decay over time. The objective of this study was to explore potential nonlinear change in the risk of untreated decay in children receiving caries prevention.

**Results:**

Across all study participants, there was a significant increase in the odds of untreated tooth decay over time (OR = 1.90, 95% CI 1.51, 2.39), but the rate of this risk rapidly decreased with each observational visit (OR = 0.87, 95% CI 0.93, 0.91). Overall effects substantially depended on the oral health status of participants at baseline: for children with untreated decay at their first observation, the odds of untreated decay over time was 0.39 (95% CI 0.27, 0.55). A quadratic change for this subpopulation showed that the per-visit decrease in decay was attenuated with each subsequent observation (OR = 1.12, 95% CI 1.04, 1.20).

## Introduction

Dental caries (tooth decay), a bacterial infection of the tooth enamel or dentin, is the most prevalent and preventable global childhood disease [1, 2]. Untreated dental caries affects over 20% of US school-aged children, exceeding 70% amongst low-income and minority children [3–5]. For many in these high-risk populations, access to dental care is limited due to financial, cultural, or geographic barriers [6]. To increase access to dental services and reduce oral health inequities, multiple organizations including the American Dental Association and the Centers for Disease Control and Prevention recommend school-based caries prevention programs as a supplement to traditional office-based care [4, 7–9]. School-based caries prevention programs are affordable, accessible approaches to treat large populations of children in need of care, but the frequency of care and the type of interventions provided are inconsistent across program. Notably, the comparative effectiveness of these school-based dental services is one of the top research priorities identified by the Institute of Medicine [10].

We previously demonstrated that a multi-component school-based caries prevention program was associated with a decreased risk of untreated decay in primary and permanent dentition [11–13]. Analyses from this study included generalized estimating equations and mixed-effects regression models, showing consistency in results. Findings demonstrated a 10% average decrease in untreated decay with each observation, indicating place-based preventive dental care can reduce caries prevalence in children. However, previous analyses did not consider potential serial correlation between study observations. Additionally, prevention over time was limited to linear change. Due to the complex etiology of dental caries as children age, more flexible functions of change over time may be appropriate. In this study, we use Markov chains for the marginal modeling of binary longitudinal data [14]. The utility of this approach allows for the modeling of the marginal probability of untreated dental caries while addressing serial dependence in repeated observations and incorporates individual random effects [15].

## Main text

### Methods

Data were derived from a previously completed prospective cohort study conducted from 2004 to 2012 in primary schools from three counties in Massachusetts, United States: Lynn, Cape Cod, and Boston. The primary study population consisted of children aged 5–12 years from low-income (“Title 1”) elementary schools in these three districts. Primary inclusion criteria for analysis were any child between the ages of 5 and 12 years. All participating schools had a majority of the student population from low socioeconomic backgrounds and receiving free or reduced price lunches. The study received Institutional Review Board (IRB) approval. This current study is a continuation of the analysis of the original closed program.

### Examination and interventions

All participants received twice-yearly oral examinations followed by treatment consisting of prophylaxis, fluoride varnish painted on all teeth, glass ionomer sealants placed on all pits and fissures, and interim therapeutic restorations (ITR) placed on any carious lesions. Oral examinations were conducted in an empty, dedicated room in the school (such as an empty classroom or an auditorium). For examinations, the child was supine in a portable dental chair with the clinician positioned above the child’s head. Clinicians used a dental headlamp for visibility. Children with informed consent were treated at each observational visit as long as they were enrolled in the school.

### Data collection

Data was collected in 6 month intervals by a clinical team consisting of a licensed dentist/dental hygienist and a dental assistant. At each data collection visit, the hygienist performed oral examinations and provided the appropriate treatment while assistants recorded clinical data using a proprietary tablet-based software program. Data were collected on all teeth and tooth surfaces for decayed, missing, or filled diagnoses and the treatments applied to each surface. Following data collection in each school, data from tablet computers were securely uploaded to a Data Coordinating Center and transmitted to investigators for analysis.

### Calibration and standardization

To minimize inter-examiner variability in caries diagnosis and data collection, clinicians and dental assistants were standardized and calibrated. Clinicians independently examined ten study participants at baseline and discussed caries presence. Personnel were then calibrated by independently examining a further ten participants and comparing caries diagnoses (k = 0.75) when compared to the gold standard examiner. For each year of the study, clinicians were re-standardized but not re-calibrated. Prior to participating in the program all hygienists were trained to use Fuji IX glass ionomer capsules using identical protocols to standardize care delivery.

### Outcome measures

The primary outcome for this study was untreated cavitated lesions on any tooth. Diagnosis of cavitated lesions were made based on visual-tactile oral examination and using the *Diagnostic Criteria and Procedures* for oral health surveys [16]. Advanced lesions were defined as gross cavitation. Early lesions on pits and fissures were similarly based on published diagnostic criteria and included: the explorer catching after insertion and either softness at base of the area or opacity adjacent to the area (or both). Smooth surfaces were defined as carious if either decalcification or a white spot was present. All teeth with questionable lesions, such as colored fissures, were graded as non-carious. Indicators for the status of every tooth surface were collected. Any tooth or tooth surface with untreated decay was used to identify the overall prevalence of untreated decay. The total number of teeth with cavitated lesions was computed for each participant.

### Covariates

In addition to primary clinical indicators, data were collected from each participant for age at each observation, sex, whether the subject had received prior dental care, presence of untreated decay at baseline, and the number of observations for each participant (the number of times care was received in 6 month intervals).

### Statistical analysis

Descriptive data for outcomes and covariates were presented as means/standard deviations or the number/percentage of the total sample. Prevalence of untreated decay by dentition type was analyzed using binary Markov chains with a second order serial dependence structure. This approach uses a traditional parametric model for the marginal distribution of untreated decay while also utilizing a stochastic model for individual (child) response profiles. Additionally, it allows for autocorrelation, unequal follow-up time across individuals, and missing data. Binary Markov models relax the traditional independence assumption of generalized linear mixed models [14, 15]. Primary models for this study included variables for linear and quadratic time, as well as interaction effects between time and baseline untreated decay. Models further adjusted for age and gender (race/ethnicity was unavailable). Predicted probabilities of untreated decay by dentition were estimated. Data analyses were performed using R v3.1.1. Statistical significance was set at p < 0.05.

### Results

For the analytic sample (N = 5327), the overall prevalence of untreated decay (all dentition) at baseline was 32.1% (Table 1). Approximately 8% of participants had untreated decay on adult teeth. The average baseline age of participants was 7.3 years (SD = 1.7). The sample was equally split between males and females. There was an increase in decay across all dentition between baseline and first follow-up, after which prevalence stabilized (Table 2).

**Table 1:** Sample descriptive statistics at baseline (N = 5327)

	N	%
Age (mean/SD)	7.25	1.69
Males	2693	50.55
Previous dental care	3187	59.83
Decay (all teeth)	1712	32.14
Decay (primary teeth)	1525	28.63
Decay (permanent teeth)	424	7.96

**Table 2:** Prevalence of untreated decay by visit and dentition

	Total	All teeth		Primary teeth		Permanent teeth	
	N	N	%	N	%	N	%
Visit							
0	5327	1712	32.14	1525	28.63	424	7.96
1	2706	924	34.15	823	30.41	277	10.24
2	1827	594	32.51	531	29.06	169	9.25
3	850	297	34.94	270	31.76	83	9.76
4	440	151	34.32	138	31.36	36	8.18

Model results (Table 3) suggest that for children without baseline decay, there was a significant increase in the odds of untreated decay over time for all dentition (OR = 1.90, 95% CI 1.51, 2.39), primary dentition (OR = 1.31, 95% CI 1.13, 1.51), and permanent dentition (OR = 1.31, 95% CI 1.15, 1.50). Quadratic time was similarly significant for all dentition types, showing a rapidly decreasing risk of decay over time (OR = 0.87, 95% CI 0.83, 0.91 for any dentition). Interaction effects with baseline decay were also significant, suggesting that prevention was more impactful in children with baseline decay, with a large significant reduction in the odds of decay over time (OR = 0.39, 95% CI 0.27, 0.55). The interaction between quadratic time and baseline decay was significant: the decrease in decay risk over time diminished with each 6-month interval (OR = 1.12, 95% CI 1.04, 1.20).

**Table 3:** Model results for untreated decay by dentition: time and baseline decay

Variable	All dentition			Primary dentition			Permanent dentition		
	OR	95% L	95% U	OR	95% L	95% U	OR	95% L	95% U
Time	1.90	1.51	2.39	1.31	1.13	1.51	1.31	1.15	1.50
Time^2^	0.87	0.83	0.91	0.95	0.92	0.97	0.95	0.92	0.97
Time*Baseline decay	0.39	0.27	0.55	0.41	0.34	0.50	0.45	0.37	0.54
Time^2^*Baseline decay	1.12	1.04	1.20	1.11	1.07	1.15	1.08	1.04	1.11

Model dependence	Coef	SE	Coef	SE	Coef	SE			
ψ_1_	1.820	0.085	2.370	0.049	2.093	0.045			
ψ_2_	1.417	0.108	0.899	0.068	0.923	0.060			

Results shown for selected covariates only; confounders (e.g., sex, baseline decay) not shown

### Discussion

School-based caries prevention can improve oral health by increasing access to care for high-risk populations [17, 18]. However, comparative approaches to prevention have shown that impacts on oral health are inconsistent, and the optimal mix of interventions, frequency of care, and program duration is still unknown [12]. In this study, we demonstrated that previous findings on the potential impact of comprehensive caries prevention were robust to alternative correlational structures, such as serial dependence, which may be a more realistic assumption when using longitudinal caries data. Additionally, as school-based caries prevention data is typically characterized by variable rates of follow-up across individuals, it is particularly well suited to this approach [14]. We also showed that effects were not linear over time for children regardless of their baseline decay status.

For children without decay at baseline, the odds of subsequent untreated decay increased over time. By definition, total decay prevalence in these children is restricted to either stay the same or increase, and thus this finding is not surprising. However, significant quadratic effects showed that this increase slowed with each observational visit, suggesting that continued preventive care may be beneficial regardless of baseline decay status. When isolated in those children with pre-existing decay at baseline, the reverse was true: there was a significant per-visit linear decrease in the odds of untreated decay, but this decrease would reduce in magnitude with each observation. This suggests that there is an immediate positive benefit of prevention to children with unmet dental needs, but prevention becomes less effective over time.

The United States Community Preventive Services Task Force recommends sealants as the primary preventive method for caries programs [18, 19]. Overall, dental sealant programs prevent subsequent tooth fillings and were found to be cost-effective [19]. However, as a major contributor to poor oral health in high-risk children is a lack of access to care, providing dental services to children in the form of a school-based program need not be limited to sealants alone. Fluoride varnish can support the prevention of dental caries in children and adolescents as shown in systematic reviews, and can be provided in tandem with dental sealants [20]. Further, despite the preventive benefit of these treatments, neither can be used to treat existing infections, which as shown in this study and others can range from 20 to 30% [4]. For many high-risk children, traditional procedures for cavity treatment are cost-prohibitive. As reported by the American Academy of Pediatric Dentistry, ITRs and atraumatic restorations are endorsed by the WHO for both restoring and preventing caries, particularly in populations that lack access to traditional dental care [21]. ITR can thus restore, arrest, and prevent caries in children in the absence of more traditional methods and have been shown to have considerably high survival rates for single-surface or multiple-surface restorations [22]. The combination of treatments can form a comprehensive prevention program that treats existing infection and prevents the spread or incidence of caries; however, the additional time and costs required is a consideration for any school-based program. Alternative agents such as silver diamine fluoride (SDF) can be safely and efficiently used at a fraction of the cost and time of ITRs, however research in large pragmatic settings is ongoing [23, 24].

## Limitations

- Despite consistency of results across different correlation structures, results may be biased due to missing data from loss to follow-up. A substantial number of study participants received a single treatment, and were thus unable to be included in analysis. If these participants had differential responses to treatment, the preventive effect may be biased.
- Data for potential confounders such as socioeconomic status and participant ethnicity were not available. Notably, previous analyses of this data showed that school-level race/ethnicity and student socioeconomic status (SES) were not significantly related to untreated caries [13].
- As an open cohort study in which all students received care, there was no control group. As such, effects cannot be considered causal.

## Data Availability

The datasets used and/or analyzed during the current study are available from the corresponding author on reasonable request.

## Abbreviations

IRB: Institutional Review Board
ITR: interim therapeutic restoration
SDF: silver diamine fluoride
SES: socioeconomic status
